# Prediction of autonomic dysreflexia during urodynamics: a prospective cohort study

**DOI:** 10.1186/s12916-018-1040-8

**Published:** 2018-04-13

**Authors:** Matthias Walter, Stephanie C. Knüpfer, Jacquelyn J. Cragg, Lorenz Leitner, Marc P. Schneider, Ulrich Mehnert, Andrei V. Krassioukov, Martin Schubert, Armin Curt, Thomas M. Kessler

**Affiliations:** 1 Neuro-Urology, Spinal Cord Injury Center & Research, University of Zürich, Balgrist University Hospital, Zürich, Switzerland; 2 Neurology, Spinal Cord Injury Center & Research, University of Zürich, Balgrist University Hospital, Zürich, Switzerland; 3 International Collaboration On Repair Discoveries (ICORD), Faculty of Medicine, University of British Columbia, Vancouver, Canada

**Keywords:** Autonomic dysreflexia, Neurogenic detrusor overactivity, Neurogenic lower urinary tract dysfunction, Prediction, Spinal cord injury, Urodynamic investigation

## Abstract

**Background:**

Autonomic dysreflexia is a severe and potentially life-threatening condition in patients with spinal cord injury, as it can lead to myocardial ischemia, brain hemorrhage, or even death. Urodynamic investigation is the gold standard to assess neurogenic lower urinary tract dysfunction due to spinal cord injury and reveal crucial pathological findings, such as neurogenic detrusor overactivity. However, neurogenic detrusor overactivity and urodynamic investigation are known to be leading triggers of autonomic dysreflexia. Therefore, we aimed to determine predictors of autonomic dysreflexia in individuals with spinal cord injury during urodynamic investigation.

**Methods:**

This prospective cohort study included 300 patients with spinal cord injuries and complete datasets of continuous non-invasive cardiovascular monitoring, recorded during same session repeat urodynamic investigation. We used logistic regression to reveal predictors of autonomic dysreflexia during urodynamic investigation.

**Results:**

We found that level of injury and presence of neurogenic detrusor overactivity were the only two independent significant predictors for autonomic dysreflexia during urodynamic investigation. A lesion at spinal segment T6 or above (odds ratio (OR) 5.5, 95% CI 3.2–9.4) compared to one at T7 or below, and presence of neurogenic detrusor overactivity (OR 2.7, 95% confidence interval (CI) 1.4–4.9) were associated with a significant increased odds of autonomic dysreflexia during urodynamic investigation. Both odds persisted after adjustment for age, sex, and completeness and stage of injury (adjusted OR (AOR) 6.6, 95% CI 3.8–11.7, and AOR 2.2, 95% CI 1.1–4.5, respectively). Further stratification by lesion level showed level-dependent significantly increased adjusted odds of autonomic dysreflexia, i.e., from C1–C4 (AOR 16.2, 95% CI 5.9–57.9) to T4–T6 (AOR 2.6, 95% CI 1.3–5.2), compared to lesions at T7 or below.

**Conclusions:**

In patients with neurogenic lower urinary tract dysfunction due to spinal cord injury, autonomic dysreflexia is independently predicted by lesion level and presence of neurogenic detrusor overactivity. Considering the health risks associated with autonomic dysreflexia, such as seizures, stroke, retinal bleeding, or even death, we recommend both continuous cardiovascular monitoring during urodynamic investigation in all spinal cord-injured patients with emphasis on those with cervical lesions, and appropriate neurogenic detrusor overactivity treatment to reduce the probability of potentially life-threatening complications.

**Trial registration:**

ClinicalTrials.gov, NCT01293110.

**Electronic supplementary material:**

The online version of this article (10.1186/s12916-018-1040-8) contains supplementary material, which is available to authorized users.

## Background

Spinal cord injury (SCI), a devastating event [1], is acquired by almost half a million people each year worldwide [2]. Besides motor recovery, i.e., the ability to walk or utilize the upper extremities, lower urinary tract (LUT) [3, 4] and cardiovascular system (CVS) [5] function are among the primary priorities for patients with SCI [6]. Autonomic dysreflexia (AD) [7], a severe, potentially life-threatening condition affecting the CVS following SCI that can occur more than 40 times a day [8], is elicited by either noxious (e.g., pain) and innocuous stimuli (e.g., bladder filling) from below the level of injury [9]. If misdiagnosed or poorly managed, AD can result in disastrous consequences [10], including myocardial ischemia [11], brain hemorrhage [12], seizures [13], and even death [14].

Urodynamic investigation (UDI) is the gold standard to assess neurogenic lower urinary tract dysfunction (NLUTD) in patients with SCI [3, 4], but it may induce AD [15, 16]. Considering that it is currently not possible to know in advance which patient will experience AD during UDI and, of those with AD, to what extent the systolic blood pressure (SBP) will increase, we aimed to determine the overall incidence and predictors of AD during UDI.

## Methods

### Participants and study design

This prospective cohort study was conducted at a single university SCI center between January 2011 and December 2016. Inclusion criteria were patients with NLUTD due to suprasacral SCI and age of at least 18 years. Exclusion criteria were symptomatic urinary tract infections (i.e., positive urine culture and clinical symptoms including fever), pressure ulcers at the time of UDI, and incomplete data of cardiovascular monitoring (CVM) during UDI. Overall, 300 patients (41 females and 259 males, mean age 51 ± 16 years, mean duration since SCI 10 ± 12 years) with complete cardiovascular recordings were included for analysis. The patients’ characteristics are shown in Table 1. This study was approved by the local ethics committee and is registered at ClinicalTrials.gov (NCT01293110). All patients gave written informed consent according to the Helsinki II declaration.

**Table 1:** Patients’ characteristics and cardiovascular changes

Characteristics	All patients (*n* = 300)	Female (*n* = 41)	Male (*n* = 259)	*p* value
Mean (SD) age (years)	51 (16)	57 (18)	51 (16)	**0.016**
Mean (SD) time after SCI (years)	10 (12)	6 (10)	11 (12)	**0.019**
Stage of SCI^a^				
Acute vs. chronic, no. (%)	68 (23) vs. 232 (7)	16 (39) vs. 25 (61)	52 (20) vs. 207 (80)	**0.007**
Type of plegia				
Tetraplegic vs. paraplegic, no. (%)	98 (33) vs. 202 (67)	11 (27) vs. 30 (73)	87 (45) vs. 172 (65)	0.391
Completeness of lesion (AIS)				
Complete (AIS A) vs. incomplete (AIS B-D), no. (%)	120 (40) vs. 180 (60)	9 (22) vs. 32 (78)	111 (43) vs. 148 (57)	**0.011**
Motor complete (AIS A–B) vs. incomplete (AIS C–D), no. (%)	170 (57) vs. 130 (43)	15 (37) vs. 26 (63)	155 (60) vs. 104 (40)	**0.005**
AIS A, no. (%)	120 (40)	9 (22)	111 (43)	
AIS B, no. (%)	50 (17)	6 (15)	44 (17)	
AIS C, no. (%)	45 (15)	9 (22)	36 (14)	
AIS D, no. (%)	85 (28)	17 (41)	68 (26)	
Lesion level				
At or above T6 vs. below T6, No. (%)	166 (55) vs. 134 (45)	16 (39) vs. 25 (61)	150 (58) vs. 109 (42)	**0.024**
Cervical, no. (%)	98 (33)	11 (27)	87 (33)	
Thoracic, no. (%)	172 (57)	20 (49)	152 (59)	
Lumbar (L1–L2), no. (%)	30 (10)	10 (24)	20 (8)	
Start of UDI^b^				
Blood pressure				
Mean (SD) systolic (mmHg)	127 (23)	123 (24)	127 (23)	0.237
Mean (SD) diastolic (mmHg)	76 (13)	71 (15)	76 (13)	**0.010**
Mean (SD) heart rate (bpm)	75 (15)	76 (14)	75 (16)	0.956
Cardiovascular change (Δ) during UDI^b^				
Blood pressure				
Mean (SD) systolic (mmHg)	42 (34)	38 (22)	43 (35)	0.384
Mean (SD) diastolic (mmHg)	17 (14)	18 (12)	17 (14)	0.697
Mean (SD) heart rate (bpm)	–8 (14)	–5 (10)	–8 (15)	0.086
Presence of NDO^b^				
Yes vs. No, no. (%)	249 (83) vs. 51 (17)	34 (83) vs. 7 (17)	215 (83) vs. 44 (17)	0.989

Statistically significant differences (p<0.05) between female and male patients are highlighted in bold

All values are presented as mean (SD) or number of patients (%)

^a^SCI defined as “acute” upon 300 days since injury and “chronic” after 300 days according to the European Multicenter Study about Spinal Cord Injury (EMSCI, www.emsci.org)

^b^Indicating the worse of two same session UDIs

*AIS* American Spinal Injury Association (ASIA) Impairment Scale, *NDO* neurogenic detrusor overactivity, *SCI* spinal cord injury, *SD* standard deviation, *UDI* urodynamic investigation

### Predictor variables and outcomes

The neurological level and completeness of SCI, i.e., sensorimotor impairment, were determined using the American Spinal Injury Association Impairment Scale (AIS) according to the International Standards for Neurological Classification of Spinal Cord Injury (ISNCSCI) [17]. The stage of injury was classified as acute (less than 300 days since injury) and chronic (more than 300 days since injury), in accordance with the European Multicenter Study about Spinal Cord Injury (EMSCI, www.emsci.org) definition.

All methods, definitions, and units are in line with the standards recommended by the International Continence Society (ICS) [18, 19]. All patients were asked to empty their bladder and bowels prior to the UDI, which was performed according to Good Urodynamic Practice following the recommendations of the ICS [18].

Individuals were investigated in a sitting position, whenever possible, which reflects the position of an individual with SCI when emptying the bladder, e.g., using intermittent self-catheterization, while sitting in a wheelchair or on a toilet. A 7 French transurethral latex-free single-use catheter and a common rectal catheter for simultaneous measurements of vesical and abdominal pressure were used. Since rectal manipulation itself can elicit AD [20], UDI was only initiated in the absence of AD. The bladder was filled retrograde with a 37°C mixture of 0.9% sodium chloride solution and contrast medium.

All patients underwent same session repeat UDI [21], i.e., two consecutive measurements, using a multichannel urodynamic system (Sedia®, Givisiez, Switzerland). Continuous cardiovascular monitoring (Finometer® PRO, Finapres Medical Systems (FMS), Amsterdam, The Netherlands) [22] was applied to enable a non-stop “beat-to-beat” documentation of SBP, diastolic BP (DBP), and heart rate (HR) synchronous to the ongoing UDI. AD (the primary outcome) was defined according to the International Standards to document remaining Autonomic Function after SCI (ISAFSCI) as an increase in SBP ≥ 20 mmHg from baseline [23]. In case of clinical signs of AD such as headache, flushing, sweating, and piloerection [24, 25], UDI was stopped immediately and the bladder was emptied.

### Statistical analysis

Continuous variables including age, time since injury, SBP, DBP, and HR were visually inspected for normal distribution by using Q-Q plots. Normally distributed data were analyzed using analysis of variance (ANOVA) or unpaired *t* tests to compare between groups. Results are presented as mean ± standard deviation (SD) or 95% confidence intervals (CIs), respectively. The *k* statistic was used to investigate agreement of the presence or absence of AD between the two UDI sessions. A chi-square test or Fisher’s exact test were used to assess the relationship between categorical variables.

Logistic regression (bivariable and multivariable) was used to investigate predictors of AD during UDI. Unadjusted odds ratios (ORs) and adjusted odds ratios (AORs) are presented with corresponding 95% CI. Statistical significance was defined as a *p* value of less than 0.05. Statistical analyses were performed using R Studio version 1.0.136 (Integrated Development for R, RStudio, Inc., Boston, MA, USA).

## Results

### Overall incidence and repeatability of autonomic dysreflexia during urodynamic investigation

**Fig. 1:**
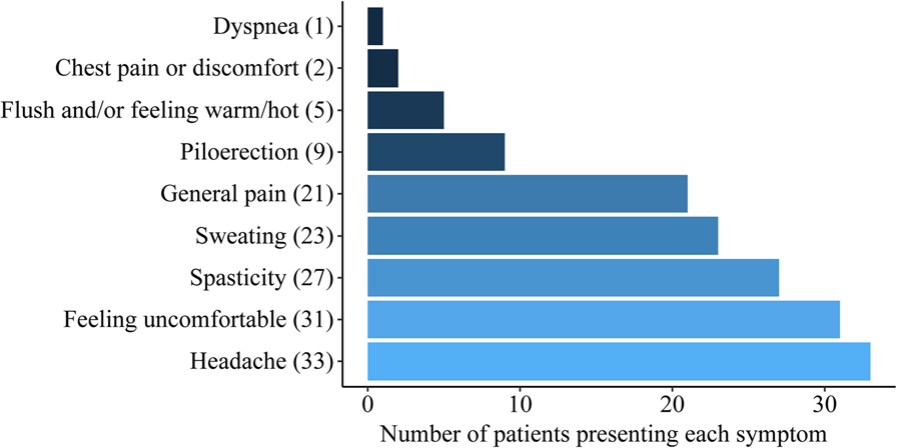
Characteristics of clinical symptoms related to autonomic dysreflexia (AD) during urodynamic investigation. In 75 patients with symptomatic AD, 152 counts of clinical symptoms were recorded. Headache with 33 counts was the most frequent symptom. The remaining 119 counts included feeling uncomfortable (31), spasticity (27), sweating (23), general pain (21), piloerection (9), flushing and/or feeling warm/hot (5), chest pain or discomfort (2), and dyspnea (1)

The overall incidence of AD during UDI was 68% (204/300). AD was found in 176 (59%) vs. 162 (54%) of the 300 patients during the first and the second UDI, respectively. In 66% (134/204) of patients with AD, an increase in SBP of at least 20 mmHg was elicited during both UDIs. Hence, in more than one third of all patients with AD (70/204), bladder filling elicited AD in only one of two UDIs. The repeatability of detecting AD between the two same session UDIs was moderate (*k* = 0.53, 95% CI –0.4 to 1.5). Characteristics of clinical symptoms presented by 37% (75/204) of patients with AD during UDI are shown in Fig. 1. We did not observe any imminent complication as a result of the increase in SBP associated with AD during UDI. All patients were monitored until SBP returned to baseline values and, whenever present, clinical symptoms disappeared.

### Prediction of autonomic dysreflexia during urodynamic investigation

**Fig. 2:**
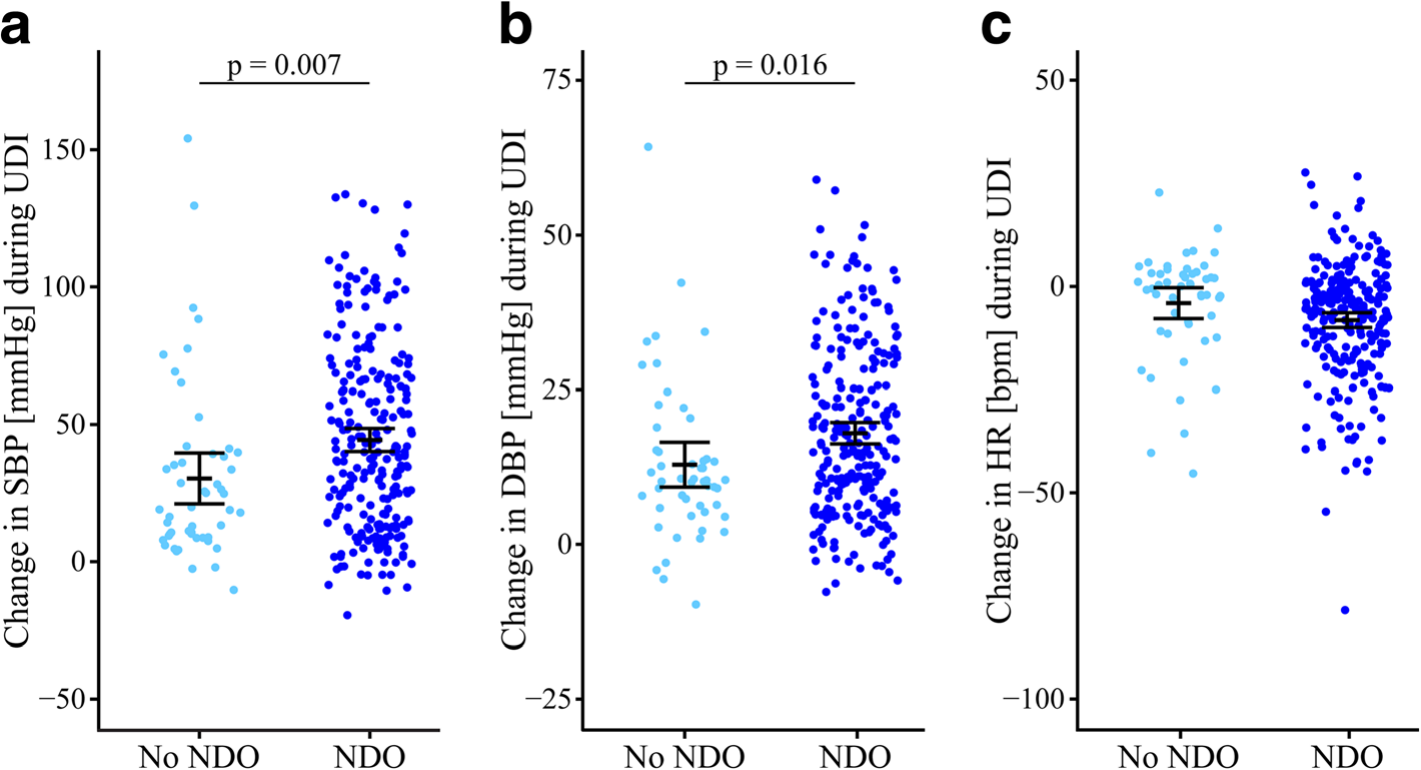
Cardiovascular parameters during urodynamic investigation by presence of neurogenic detrusor overactivity. Cardiovascular changes in (**a**) SBP, (**b**) DBP, and (**c**) HR in SCI patients who have either presented NDO (*right side in dark blue*) or not (*left side in Maya blue*). At the start of UDI, cardiovascular parameters were not significantly different between both groups. In patients with NDO, cardiovascular changes in SBP (44, 95% CI 40.2–48.6 vs. 30, 95% CI 21.1–39.6 mmHg, *p* = 0.007) and DBP (18, 95% CI 16.3–19.8 vs. 13, 95% CI 9.3–16.6 mmHg, *p* = 0.016) during UDI were significantly different compared to patients without NDO. Changes in HR (–8, 95% CI –10.0 to –6.4 vs. −4, 95% CI –7.7 to –0.4) did not significantly (*p* = 0.06) differ between both groups. Each *circle* represents one patient’s cardiovascular changes during UDI. Error bars represent mean and the 95% CI of cardiovascular changes; i.e., the worse out of two same session UDIs was used. *BPM* beats per minute, *CI* confidence interval, *DBP* diastolic blood pressure, *HR* heart rate, *NDO* neurogenic detrusor overactivity, *SBP* systolic blood pressure, *SCI* spinal cord injury, *UDI* urodynamic investigation

Logistic regression analyses (Table 2) revealed that the lesion level, i.e., T6 or above vs. T7 or below, as well as the presence of neurogenic detrusor overactivity (NDO) predicted AD during UDI. Further stratification by lesion level showed a significantly increasing odds of AD with higher lesion levels. When comparing patients with NDO and without NDO, statistically significant increases in SBP and DBP were observed, while changes in HR did not significantly differ between both groups (Fig. 2).

**Table 2:** Odds ratios for autonomic dysreflexia during urodynamics

Variable	Unadjusted OR (95% CI)	*p* value	Adjusted OR (95% CI)	*p* value
Sex				
Male^a^	1		1	
Female	2.1 (1.0–5.1)	0.070	2.0 (0.9–4.9)	0.101
Stage of SCI^b^				
Acute	1.2 (0.7–2.1)	0.600	0.8 (0.4–1.7)	0.622
Chronic^a^	1		1	
AIS				
A	0.9 (0.5–1.6)	0.719	1.4 (0.7–2.9)	0.700
B	1.2 (0.6–2.6)	0.646	1.7 (0.7–4.3)	0.360
C	1.0 (0.5–2.3)	0.939	1.2 (0.5–2.9)	0.850
D^a^	1		1	
Complete vs. incomplete SCI				
AIS A–B	0.9 (0.5–1.4)	0.510	1.1 (0.7–2.0)	0.657
AIS C–D^a^	1		1	
Motor complete vs. incomplete SCI				
AIS A	1.0 (0.6 – 1.6)	0.880	1.4 (0.8 – 2.6)	0.250
AIS B-D^a^	1		1	
Lesion level, cutoff at T6				
At or above T6	5.5 (3.2–9.4)	**< 0.001**	6.6 (3.8–11.7)	**< 0.001**
T7 and below^a^	1		1	
Lesion level, distributions above T7				
C1–C4	13.9 (5.2–48.8)	**< 0.001**	16.2 (5.9–57.9)	**< 0.001**
C5–C8	8.5 (3.5–23.8)	**< 0.001**	12.2 (4.9–35.8)	**< 0.001**
T1–T3	4.3 (1.8–11.7)	**0.002**	5.2 (2.1–14.5)	**0.001**
T4–T6	2.4 (1.3–4.7)	**0.007**	2.6 (1.3–5.2)	**0.006**
T7 and below^a^	1		1	
Presence of NDO during UDI^c^				
Yes	2.7 (1.4–4.9)	**0.002**	2.2 (1.1–4.5)	**0.030**
No^a^	1		1	

Statistically significant differences (p<0.05) for the categorical variables against its own reference category are highlighted in bold

All values are presented as OR (95% CI)

Bivariate logistic regression analysis between AD and each categorical variable resulted in crude OR. Multivariate logistic regression analysis between AD and all categorical variables including age (as a continuous variable) in one model resulted in adjusted OR

^a^Reference category

^b^SCI defined as “acute” upon 300 days since injury and “chronic” after 300 days according to the European Multicenter Study about Spinal Cord Injury (EMSCI, www.emsci.org)

^c^Indicating the worse of two same session UDIs

*AD* autonomic dysreflexia, *AIS* American Spinal Injury Association (ASIA) Impairment Scale, *C* cervical, *CI* confidence interval, *NDO* neurogenic detrusor overactivity, *OR* odds ratio, *SCI* spinal cord injury, *T* thoracic, *UDI* urodynamic investigation

### Differences between patients with and without autonomic dysreflexia

When comparing patients with AD and without AD, statistically significant differences in SBP, DBP, and HR were observed (Table 3). Remarkably, almost one third (65/204, 32%) of the patients with AD had a lesion below T6.

**Table 3:** Baseline characteristics and cardiovascular changes: differences between patients with and without AD

Characteristics	No AD (*n* = 96)	AD (*n* = 204)	*p* value
Sex			
Male vs. female, no. (%)	88 (92) vs. 8 (8)	171 (84) vs. 33 (16)	0.065
Mean (SD) age (years)	49 (15)	53 (16)	**0.044**
Mean (SD) time after SCI (years)	10 (10)	11 (12)	0.706
Stage of SCI^a^			
Acute vs. chronic, no. (%)	20 (21) vs. 76 (79)	48 (23) vs. 156 (77)	0.603
Type of plegia			
Tetraplegic vs. paraplegic, no. (%)	10 (10) vs. 86 (90)	88 (43) vs. 116 (57)	**< 0.001**
Completeness of lesion (AIS)			
Complete (AIS A) vs. incomplete (AIS B–D), no. (%)	41 (43) vs. 55 (57)	79 (39) vs. 125 (61)	0.511
Motor complete (AIS A–B) vs. incomplete (AIS C–D), no. (%)	55 (57) vs. 41 (43)	115 (56) vs. 89 (44)	0.881
AIS A, no. (%)	41 (43)	79 (39)	
AIS B, no. (%)	14 (15)	36 (18)	
AIS C, no. (%)	14 (15)	31 (15)	
AIS D, no. (%)	27 (28)	58 (28)	
Lesion level			
At or above T6 vs. below T6, no. (%)	27 (28) vs. 69 (72)	139 (68) vs. 65 (32)	**< 0.001**
Cervical, no. (%)	10 (10)	88 (43)	
Thoracic, no. (%)	72 (75)	100 (49)	
Lumbar (L1–L2), no. (%)	14 (15)	16 (8)	
Start of UDI^b^			
Blood pressure			
Mean (SD) systolic (mmHg)	133 (22)	124 (23)	**0.001**
Mean (SD) diastolic (mmHg)	79 (15)	74 (12)	**0.001**
Mean (SD) heart rate (bpm)	74 (15)	76 (16)	0.458
Cardiovascular change (Δ) during UDI^b^			
Blood pressure			
Mean (SD) systolic (mmHg)	7 (8)	58 (29)	**< 0.001**
Mean (SD) diastolic (mmHg)	5 (6)	23 (13)	**< 0.001**
Mean (SD) heart rate (bpm)	1 (8)	–11 (15)	**< 0.001**
Presence of NDO^b^			
Yes vs. No, no. (%)	70 (73) vs. 26 (27)	179 (88) vs. 25 (12)	**0.001**

Statistically significant differences (p<0.05) between patients with and without autonomic dysreflexia are highlighted in bold

All values are presented as mean (SD) or number of patients (%)

^a^SCI defined as “acute” upon 300 days since injury and “chronic” after 300 days according to the European Multicenter Study about Spinal Cord Injury (EMSCI, www.emsci.org)

^b^Indicating the worse of two same session UDIs

*AD* autonomic dysreflexia, *AIS* American Spinal Injury Association (ASIA) Impairment Scale, *NDO* neurogenic detrusor overactivity, *SCI* spinal cord injury, *SD* standard deviation, *UDI* urodynamic investigation

### Characteristics of spinal cord injury affect cardiovascular changes in patients with autonomic dysreflexia

**Fig. 3:**
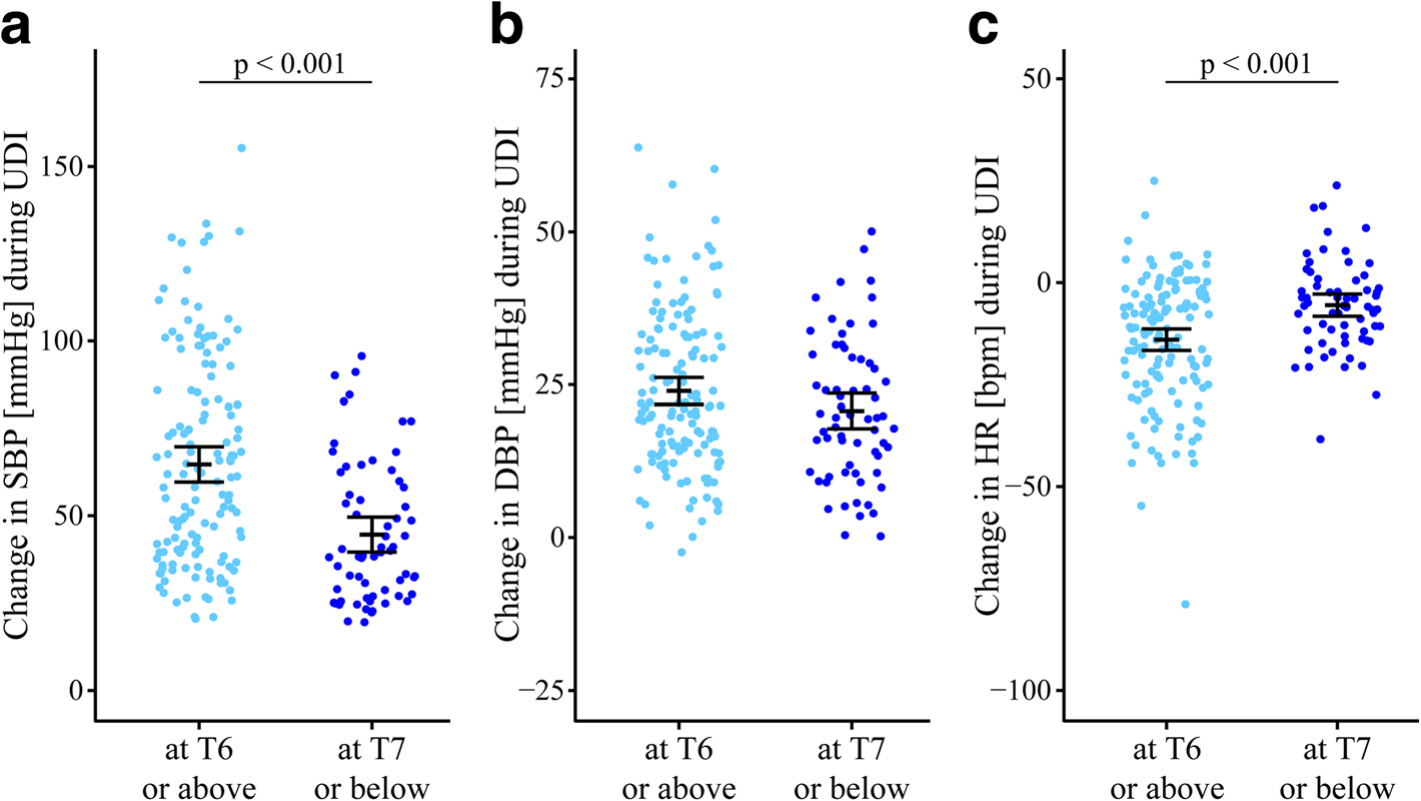
Cardiovascular changes during urodynamic investigation by lesion level (cutoff T6). Cardiovascular changes in (**a**) SBP, (**b**) DBP, and (**c**) HR in SCI patients with AD who have either sustained a SCI at T6 or above (*left side in Maya blue*) or at T7 or below (*right side in dark blue*). At the start of UDI, cardiovascular parameters were not significantly different between both groups. In patients with a SCI at T6 or above, cardiovascular changes in SBP (65, 95% CI 59.7–69.7 vs. 45, 95% CI 39.6–49.5 mmHg) and HR (−14, 95% CI –16.6 to –11.4 vs. −6, 95% CI –8.2 to –2.9 bpm) were significantly different (*p* < 0.001) compared to those of remaining patients. Each *circle* represents one patient’s cardiovascular changes during UDI. Error bars represent mean and the 95% CI of cardiovascular changes; i.e., the worse out of two same session UDIs were used. *AD* autonomic dysreflexia, *BPM* beats per minute, *CI* confidence interval, *DBP* diastolic blood pressure, *HR* heart rate, *SBP* systolic blood pressure, *SCI* spinal cord injury, *T* thoracic, *UDI* urodynamic investigation

**Fig. 4:**
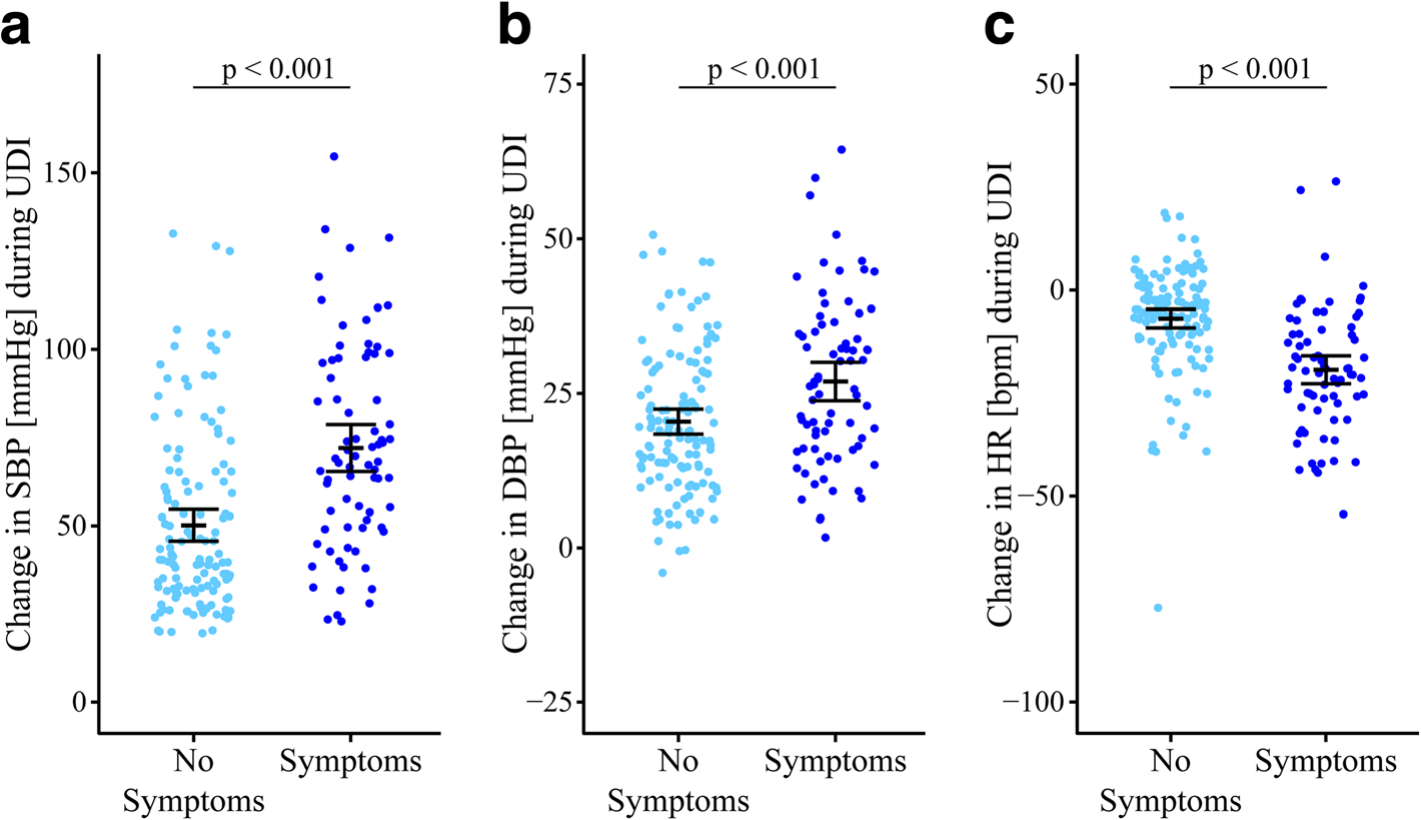
Cardiovascular parameters during urodynamic investigation by symptomatic vs. asymptomatic AD. Cardiovascular changes in (**a**) SBP, (**b**) DBP, and (**c**) HR in SCI patients with AD who have either presented symptoms (*right side in dark blue*) or not (*left side in Maya blue*). At the start of UDI, cardiovascular parameters were not significantly different between both groups. In patients with symptomatic AD, cardiovascular changes in SBP (72, 95% CI 67.6–76.7 vs. 50, 95% CI 45.8–54.8 mmHg), DBP (27, 95% CI 25.0–29.1 vs. 21, 95% CI 18.5–22.6 mmHg) and HR (−19, 95% CI –21.6 to –16.7 vs. −7, 95% CI –9.0 to –4.3) during UDI were significantly different (p < 0.001) compared to those of remaining patients. Each *circle* represents one patient’s cardiovascular changes during UDI. Error bars represent mean and the 95% CI of cardiovascular changes; i.e., the worse out of two same session UDIs were used. *AD* autonomic dysreflexia, *BPM* beats per minute, *CI* confidence interval, *DBP* diastolic blood pressure, *HR* heart rate, *SBP* systolic blood pressure, *SCI* spinal cord injury, *UDI* urodynamic investigation

Exploring cardiovascular changes during UDI within the group of patients with AD, we found several significant differences related to SCI severity, i.e., the level and the completeness of the lesion, and the presence of clinical symptoms. Specifically, patients with cervical lesions experienced a significantly higher increase in SBP during UDI compared to those with a thoracic and lumbar lesion (Additional file 1). Patients with lesions at T6 or above showed a significantly higher increase in SBP and a greater decrease in HR compared to those with a lesion at T7 or below (Fig. 3). When stratifying patients with AD according to the AIS, significantly different changes in HR were discovered between AIS A and AIS C, AIS A and AIS D, and AIS B and AIS D (Additional file 1). Patients suffering from a complete lesion (AIS A) compared to those with an incomplete lesion (AIS B–D) showed a significantly greater decrease in HR (Additional file 1). Patients with a motor complete lesion (AIS A–B) compared to those with a motor incomplete lesion (AIS C–D) had a significantly higher increase in SBP and a greater decrease in HR (Additional file 1). Patients with symptomatic compared to asymptomatic AD demonstrated a significantly higher increase in SBP and DBP as well as a greater decrease in HR (Fig. 4). No significant cardiovascular changes were found for AD patients with acute compared to those with chronic suprasacral SCI (Additional file 1).

## Discussion

In this prospective cohort study including 300 patients, we found a high incidence of AD in about two thirds of our patients with NLUTD due to suprasacral SCI. In addition, we identified the level of SCI as well as the presence of NDO as independent significant predictors to experience AD during UDI. Importantly, almost one third of our patients with AD had a lesion level below T6.

A high number of physicians and other healthcare professionals may not be aware of AD, unless their patient population includes individuals with SCI [26]. Thus, the lack of experience with AD-related SBP changes might lead to misdiagnosed AD and even acceptance of the potential risk of life-threatening complications during UDI. Providing evidence on what constitutes an increased risk for experiencing AD during UDI, i.e., predictors, could lead to a safer way to perform UDI in this cohort. Most importantly, education regarding AD, i.e., knowledge transfer to physicians, other health professionals, caregivers, and especially patients and family members is crucial to raise awareness of AD so that AD-related complications during UDI and in everyday life (including emergency room admissions for “not feeling right” or urinary tract symptoms) can be minimized or prevented at best.

In contrast to episodic BP measurement, which has often been used to record AD during UDI [27–29], continuous CVM provides a more accurate observation, as it allows one to detect short episodes of AD. The “beat-to-beat” technique allows the Finometer (via finger photoplethysmography) to monitor cardiovascular changes of SBP, DBP, and HR continuously and accurately according to the Association for the Advancement of Medical Instrumentation. Furthermore, the British Hypertension Society has recommended the Finometer for measurements in the clinical set-up and for research purposes. In line with Liu et al. [9] and our previous study [16], we used continuous CVM to document SBP, DBP, and HR, i.e., “beat-to-beat” recordings, throughout the entire investigation to immediately reveal AD. In fact, AD was detected during UDI in more than two thirds (204/300) of our patients. In contrast, previous literature measuring BP episodically reported incidence of AD between 37 and 43%. This lower incidence of AD might be attributed to intermittent instead of continuous CVM, as the former is likely to miss short-term episodes of AD [27–29]. Evidence for this hypothesis is given, as we recently found a similarly high incidence of AD (73%) in women with NLUTD due to suprasacral SCI [16]. Thus, AD during UDI in individuals with SCI seems to be generally underestimated, and this might put our patients at relevant risk if AD is not detected and appropriately managed.

Sympathetic pre-ganglionic neurons (SPNs) are regarded as the pivotal spinal neurons for central cardiovascular control [30]. It is commonly believed that AD occurs in patients with SCI at or above T6 [31, 32], and most studies have only presented data in this selected cohort [9, 27, 29, 33]. However, AD can also occur in patients with a lesion level below T6, as the sympathetic outflow originates from T1 to L2 [34] and supplies various regions, such as the heart (T1–T5) and urinary bladder (L1–L2) [35]. Thus, similar to Huang et al. [28], we also included patients with SCI below T6, and about one third of patients with AD in our study had a lesion below T6. The sympathetic postganglionic neurons, excited by SPNs, synapse with target organs, such as the heart and blood vessels, and hence are responsible for the innervation of the splanchnic vascular bed [32]. The higher the level of injury, the more SPNs are likely to be independent of central inhibition. Therefore, a growing number of independent SPNs receiving afferent input from below the level of injury could provide the critical mass of vasoconstriction needed to elevate the SBP accordingly [31]. To provide further evidence on the impact of lesion level on cardiovascular changes, increase in SBP from baseline in patients experiencing AD during UDI was highest in those with a cervical injury, followed by those with a thoracic and lumbar injury, respectively. In patients with a lesion below T6, it is thought that either sympathetic innervation of vascular structures remains sufficiently under supraspinal sympathetic control [36], or the extent of disconnected vascular areas is reduced, leading to a normal inhibitory response to baroreceptor-mediated reflexes to maintain homeostasis.

AD can also be asymptomatic, known as silent AD [29, 33]. Remarkably, 63% (129/204) of our patients with AD were asymptomatic. This condition can be very hazardous, because the discrepancy between cardiovascular changes and clinical symptoms might be misunderstood by physicians and therefore assigned incorrectly to other causes, which could lead to life-threatening situations. The group of 75 patients with symptomatic AD (37%) had significantly greater changes in all three cardiovascular domains, i.e., SPB, DBP, and HR, and consisted of more patients with a lesion level at or above T6 and complete lesions (AIS A) compared to those with asymptomatic AD.

Considering the distribution of the lesion level, i.e., at or above T6 vs. below T6, more patients who were asymptomatic had a lesion below T6. While preserved central control of SPNs allows inhibitory descending signals to counterattack the vasoconstriction, individuals with a lesion above T6 are lacking central control of SPNs that innervate the heart (T1–T5). The higher the lesion above T6, the more likely a patient will show a slowing down of the heartbeat, potentially result in bradycardia. This reflects the parasympathetic effort to respond to the sudden SBP increase in reducing the heart stroke volume through the vagal nerve. This may ultimately result in a deterioration of the patient’s condition.

In line with the literature [37], our AD patients with an AIS A lesion demonstrated a significantly greater decrease in HR than those with an AIS B–D lesion, indicating that the greater the extent of the injury, i.e., the fewer efferent fibers are spared to uphold central sympathetic control of the heart, the greater the influence on HR by the parasympathetic nervous system. In contrast to our observation, Giannantoni et al. [27] reported that AD did not correlate with the completeness of a lesion. These discrepancies might be attributed to differences in cardiovascular assessment, individual number, and characteristics of SCI, hampering a meaningful comparison but warranting further investigation of these issues. It should be acknowledged that the extent of an SCI reaches beyond the sensorimotor impairment. Whether an SCI is autonomic complete, i.e., supraspinal sympathetic control is entirely lost, or not seems to have a significant influence on blood pressure and heart rate. According to the review by West et al. [38] in patients with chronic SCI, the autonomic completeness of SCI is more strongly related to cardiovascular function than neurological completeness of injury. However, we do not have enough data from all of our patients to investigate the effect of autonomic completeness on the incidence of AD and cardiovascular changes during UDI.

In line with Liu et al. [15], we found significantly different cardiovascular changes in patients with NDO compared to those without. Being considered a major cause to trigger AD [15] and leading to significant increases in arterial pressure [39], NDO seems to pose a significant health risk to patients with SCI. This is clinically relevant, as most patients with SCI suffer from NDO [40]. As a result of NDO, repeatedly increased intravesical pressures can lead to morphological changes of the urinary tract and increased risk of upper urinary tract complications in the long term [4, 41]. The latter comprise vesico-uretero-renal reflux, hydronephrosis, impairment of renal functions, or at worst renal failure [4]. Treatment of NDO, i.e., with antimuscarinic drugs and intradetrusor onabotulinumtoxinA injections, results in reduction of intravesical pressure, improves quality of life, and has been successfully implemented into clinical practice and guidelines [3]. Given the potential of NDO to elicit AD, successful treatment of NDO could also have a positive effect on AD incidence and extent of related cardiovascular changes. In line with this hypothesis, Fougere et al. [42] provided evidence of effectively reducing the frequency and severity of AD after intradetrusor onabotulinumtoxinA injections in patients with SCI. By improving LUT function and concurrently reducing cardiovascular responses to LUT stimuli, treatment of NDO appears to have the capacity to lower the risk for AD-related long-term complications.

Although to the best of our knowledge this is the largest prospective cohort study investigating AD using continuous cardiovascular changes in patients with suprasacral SCI suffering from NLUTD, some limitations should be addressed. Common conditions such as urinary tract infections, medications, and fluctuations of the neurological state can influence lower urinary tract function and consequently UDI and CVM parameters. Furthermore, as we only included those patients with SCI who are affected by NLUTD, which represents most SCI patients, it is difficult to say whether our results could also be extrapolated to SCI patients without NLUTD. Moreover, demonstrating the impact of SCI lesion level, completeness, and symptomatology of AD on cardiovascular changes during UDI, it would be of great interest to continuously monitor cardiovascular parameters during long-term ambulatory UDI to further assess the cardiovascular risk profile in patients with SCI.

Despite these limitations, our study is the first to provide evidence that AD during UDI is predictable by lesion level and presence of NDO. Our results clearly underline the importance of continuous CVM during UDI in all patients with suprasacral SCI and emphasize the relevance of proactive NDO treatment. In this way, AD can be detected earlier and more frequently, which subsequently could lead to fewer episodes of AD-related complications during diagnostic assessment or at best none. To further protect patients with SCI, continuous CVM should also be considered as standard surveillance during other diagnostic interventions such as cystoscopy [9] and sperm retrieval [43].

## Conclusions

Considering all potential health risks associated with AD, such as seizures, stroke, retinal bleeding, or even death, we highly recommend continuous CVM during UDI in all patients with suprasacral SCI, since focusing on lesions at T6 or above would result in missing a relevant percentage of patients with AD. However, particular emphasis should be given to individuals with cervical lesions, as they are at highest risk for AD during UDI.

Given our previous experiences [16, 21], we propose performing same session repeat UDI with continuous CVM, which allows one to provide the treating urologist with more precise information on the extent of NLUTD and cardiovascular changes during UDI.

Moreover, we advise considering appropriate treatment of NDO, not only to protect the upper urinary tract from potential long-term damage, but also to improve quality of life and decrease the risk of AD. Following our recommendations will also allow for revealing findings of cardiovascular risk factors in patients with silent AD and might subsequently reduce the risk of potentially life-threating complications related to sudden hypertension during AD.

## Additional file

Additional file 1: Results. **Table S1.** Cardiovascular parameters by lesion level in patients with autonomic dysreflexia. **Table S2.** Cardiovascular parameters by completeness of injury according to the American Spinal Injury Association Impairment Scale. **Table S3.** Cardiovascular parameters by motor completeness of injury according to the American Spinal Injury Association (ASIA) Impairment Scale (AIS). **Table S4.** Cardiovascular parameters by stage of injury. **Figure S1.** Cardiovascular parameters during urodynamic investigation by American Spinal Injury Association (ASIA) Impairment Scale (AIS). (DOCX 260 kb)

## Abbreviations

AD: Autonomic dysreflexia
AIS: American Spinal Injury Association Impairment Scale
ANOVA: Analysis of variance
AOR: Adjusted odds ratio
CI: Confidence interval
CVS: Cardiovascular system
DBP: Diastolic blood pressure
EMSCI: European Multicenter Study about Spinal Cord Injury
HR: Heart rate
ICS: International Continence Society
ISAFSCI: International Standards to document remaining Autonomic Function after Spinal Cord Injury
ISNCSCI: International Standards for Neurological Classification of Spinal Cord Injury
LUT: Lower urinary tract
NDO: Neurogenic detrusor overactivity
NLUTD: Neurogenic lower urinary tract dysfunction
OR: Odds ratio
SBP: Systolic blood pressure
SCI: Spinal cord injury
SD: Standard deviation
SPN: Sympathetic pre-ganglionic neuron
UDI: Urodynamic investigation
