# Supplementary material for: Prediction of autonomic dysreflexia during urodynamics: a prospective cohort study
Source: BMC Med. 2018 Apr 13;16:53. doi: 10.1186/s12916-018-1040-8 (PMC5898013; doi:10.1186/s12916-018-1040-8)
Supplement: Supplementary file 1 — Results. Table S1. Cardiovascular parameters by lesion level in patients with autonomic dysreflexia. Table S2. Cardiovascular parameters by completeness of injury according to the American Spinal Injury Association Impairment Scale. Table S3. Cardiovascular parameters by motor completeness of injury according to the American Spinal Injury Association (ASIA) Impairment Scale (AIS). Table S4. Cardiovascular parameters by stage of injury. Figure S1. Cardiovascular parameters during urodynamic investigation by American Spinal Injury Association (ASIA) Impairment Scale (AIS). (DOCX 260 kb) [file 12916_2018_1040_MOESM1_ESM.docx]

**ADDITIONAL FILE.** Results.

**Table S1 – Cardiovascular parameters by lesion level in patients with autonomic dysreflexia.**

| **Cardiovascular parameter** | **Cervical lesions (n=88)** | **95% CI (LB)** | **95% CI (UB)** |
| --- | --- | --- | --- |
| Start of UDI^#^ |  |  |  |
| Blood pressure |  |  |  |
| Mean (SD) Systolic (mmHg) | 123 (22) | 118.6 | 127.9 |
| Mean (SD) Diastolic (mmHg) | 73 (12) | 70.6 | 75.7 |
| Mean (SD) Heart rate (bpm) | 74 (16) | 70.6 | 77.3 |
| Changes (Δ) during UDI^#^ |  |  |  |
| Blood pressure |  |  |  |
| Mean (SD) Systolic (mmHg) | 67 (32) | 60.4 | 73.9 |
| Mean (SD) Diastolic (mmHg) | 25 (14) | 21.9 | 27.8 |
| Mean (SD) Heart rate (bpm) | -12 (16) | -15.8 | -9.0 |
|  |  |  |  |
| **Cardiovascular parameter** | **Thoracic lesions (n=100)** | **95% CI (LB)** | **95% CI (UB)** |
| Start of UDI^#^ |  |  |  |
| Blood pressure |  |  |  |
| Mean (SD) Systolic (mmHg) | 124 (22) | 119.6 | 128.4 |
| Mean (SD) Diastolic (mmHg) | 75 (12) | 72.3 | 77.0 |
| Mean (SD) Heart rate (bpm) | 77 (15) | 73.7 | 79.8 |
| Changes (Δ) during UDI^#^ |  |  |  |
| Blood pressure |  |  |  |
| Mean (SD) Systolic (mmHg) | 52 (25) | 47.3 | 57.4 |
| Mean (SD) Diastolic (mmHg) | 21 (11) | 18.8 | 23.2 |
| Mean (SD) Heart rate (bpm) | -11 (14) | -14.3 | -8.6 |
|  |  |  |  |
| **Cardiovascular parameter** | **Lumbar lesion (n=16)** | **95% CI (LB)** | **95% CI (UB)** |
| Start of UDI^#^ |  |  |  |
| Blood pressure |  |  |  |
| Mean (SD) Systolic (mmHg) | 123 (28) | 108.4 | 137.9 |
| Mean (SD) Diastolic (mmHg) | 71 (16) | 62.8 | 80.1 |
| Mean (SD) Heart rate (bpm) | 81 (15) | 73.1 | 89.1 |
| Changes (Δ) during UDI^#^ |  |  |  |
| Blood pressure |  |  |  |
| Mean (SD) Systolic (mmHg) | 47 (19) | 36.9 | 57.2 |
| Mean (SD) Diastolic (mmHg) | 24 (15) | 16.1 | 32.0 |
| Mean (SD) Heart rate (bpm) | -5 (7) | -8.5 | -1.1 |

All values are presented as mean (SD) and 95% CI.

ANOVA pairwise comparisons (Bonferroni corrected) revealed significant different changes in systolic blood pressure during UDI in patients with cervical lesions compared to those with thoracic (p=0.001) and lumbar lesions (p=0.026).

# Indicating the worse of two same session UDIs.

ANOVA=analysis of variance, CI=confidence interval, LB= lower boundary, SCI=spinal cord injury, SD=standard deviation, UB= upper boundary, UDI=urodynamic investigation.

**Table S2 – Cardiovascular parameters by completeness of injury according to the American Spinal Injury Association Impairment Scale.**

| **Cardiovascular parameter** | **Complete SCI – AIS A (n=79)** | **Incomplete SCI – AIS B-D (n=125)** | ***P* Value** |
| --- | --- | --- | --- |
| Start of UDI^#^ |  |  |  |
| Blood pressure |  |  |  |
| Mean (SD) Systolic (mmHg) | 125 (24) [119.5 **–** 129.8] | 123 (22) [119.2 **–** 126.8] | 0.605 |
| Mean (SD) Diastolic (mmHg) | 74 (12) [71.3 **–** 76.5] | 74 (12) [71.5 **–** 75.9] | 0.888 |
| Mean (SD) Heart rate (bpm) | 76 (16) [72.9 **–** 80.0] | 76 (15) [72.8 **–** 78.2] | 0.682 |
| Changes (Δ) during UDI^#^ |  |  |  |
| Blood pressure |  |  |  |
| Mean (SD) Systolic (mmHg) | 60 (30) [53.7 **–** 67.0] | 57 (28) [52.0 **–** 61.9] | 0.420 |
| Mean (SD) Diastolic (mmHg) | 23 (12) [20.6 **–** 25.8] | 23 (13) [20.4 **–** 25.1] | 0.800 |
| Mean (SD) Heart rate (bpm) | -17 (15) [-20.7 **–** -14.2] | -7 (14) [-9.9 **–** -5.1] | **<0.001** |

All values are presented as mean (SD) and 95% CI.

^#^ Indicating the worse of two same session UDIs.

AIS=American spinal injury association (ASIA) impairment scale, CI=confidence interval, SCI=spinal cord injury, SD=standard deviation, UDI=urodynamic investigation.

**Table S3 – Cardiovascular parameters by motor completeness of injury according to the American Spinal Injury Association Impairment Scale.**

| **Cardiovascular parameter** | **Motor complete SCI – AIS A-B (n=115)** | **Motor incomplete SCI – AIS C-D (n=89)** | ***P* Value** |
| --- | --- | --- | --- |
| Start of UDI^#^ |  |  |  |
| Blood pressure |  |  |  |
| Mean (SD) Systolic (mmHg) | 124 (23) [120.3 **–** 128.6] | 123 (22) [118.0 **–** 127.1] | 0.546 |
| Mean (SD) Diastolic (mmHg) | 74 (12) [72.0 **–** 76.4] | 73 (12) [70.6 **–** 75.8] | 0.583 |
| Mean (SD) Heart rate (bpm) | 75 (16) [72.5 **–** 78.4] | 76 (15) [73.3 **–** 79.5] | 0.671 |
| Changes (Δ) during UDI^#^ |  |  |  |
| Blood pressure |  |  |  |
| Mean (SD) Systolic (mmHg) | 63 (32) [57.0 **–** 68.7] | 52 (23) [47.6 **–** 57.1] | **0.007** |
| Mean (SD) Diastolic (mmHg) | 23 (13) [21.0 **–** 25.8] | 22 (12) [19.7 **–** 24.9] | 0.547 |
| Mean (SD) Heart rate (bpm) | -16 (16) [-19.1 **–** -13.1] | -5 (10) [-7.2 **–** -3.1] | **<0.001** |

All values are presented as mean (SD) and 95% CI.

^#^ Indicating the worse of two same session UDIs.

AIS=American spinal injury association (ASIA) impairment scale, CI=confidence interval, SCI=spinal cord injury, SD=standard deviation, UDI=urodynamic investigation.

**Table S4 – Cardiovascular parameters by stage of injury.**

| **Cardiovascular parameter** | **Acute SCI* patients (n=48)** | **Chronic SCI* patients (n=156)** | ***P* Value** |
| --- | --- | --- | --- |
| Start of UDI^#^ |  |  |  |
| Blood pressure |  |  |  |
| Mean (SD) Systolic (mmHg) | 118 (19) [112.7 – 123.3] | 125 (23) [121.7 – 129.9] | **0.046** |
| Mean (SD) Diastolic (mmHg) | 69 (12) [66.0 – 72.7] | 75 (12) [73.2 – 77.0] | **0.004** |
| Mean (SD) Heart rate (bpm) | 79 (13) [75.5 – 82.9] | 75 (16) [72.3 – 77.4] | 0.094 |
| Changes (Δ) during UDI^#^ |  |  |  |
| Mean (SD) Systolic (mmHg) |  |  |  |
| Mean (SD) Diastolic (mmHg) | 55 (28) [47.0 – 63.0] | 59 (29) [54.8 – 63.9] | 0.363 |
| Mean (SD) Heart rate (bpm) | 21 (12) [17.7 – 24.4] | 23 (13) [21.5 – 25.5] | 0.245 |
| Mean (SD) Systolic (mmHg) | -9 (12) [-12.3 – -5.4] | -12 (16) [-14.5 – -9.6] | 0.186 |

All values are presented as mean (SD) and 95% CI.

* SCI defined as ‘acute’ upon 300 days since injury and ‘chronic’ after 300 days according to the European multicenter study about spinal cord injury (EMSCI, www.emsci.org).

^#^ Indicating the worse of two same session UDIs.

CI=confidence interval, SCI=spinal cord injury, SD=standard deviation, UDI=urodynamic investigation.


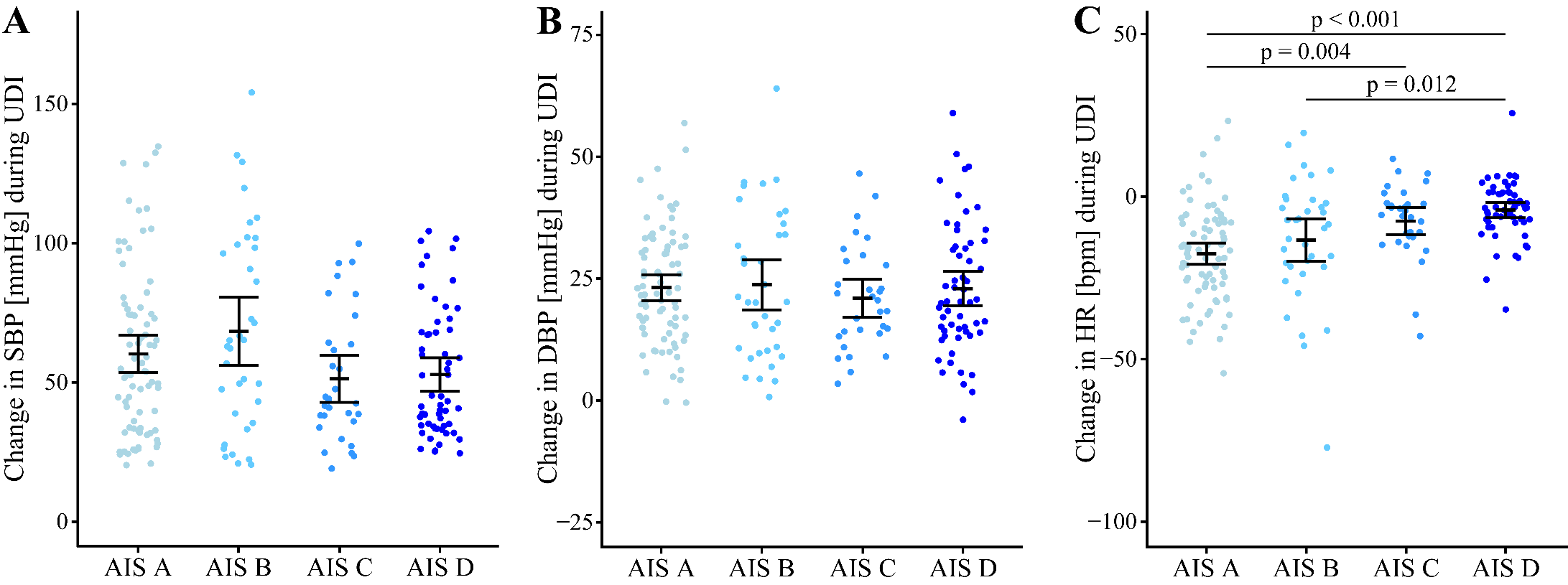


**Figure S1 – Cardiovascular parameters during urodynamic investigation by American Spinal Injury Association (ASIA) Impairment Scale (AIS).**

Cardiovascular changes in (A) SBP, (B) DBP and (C) HR in SCI patients with AD classified as AIS A (left side in light blue), AIS B (second from the left in Maya blue), AIS C (second from the right in blue), or AIS D (right side in dark blue). At the start of UDI, cardiovascular parameters were not significantly different between all groups. In patients with AD classified as AIS A, changes in HR were significantly different compared to patients classified as AIS C (-17, 95% CI -20.7 to -14.1 versus -7, 95% CI -11.5 to -3.2 bpm, p=0.004) and AIS D (-17, 95% CI -20.7 to -14.1 versus -4, 95% CI -6.3 to -1.6 bpm, p<0.001). Furthermore, in patients with AD classified as AIS B, changes in HR were significantly different compared to patients classified as AIS D (-13, 95% CI -19.8 to -6.6 versus -4, 95% CI -6.3 to -1.6 bpm, p=0.012). ANOVA - pairwise comparisons (Bonferroni corrected). Each circle represents one patient’s cardiovascular changes during UDI. Error bars are representing mean and the 95% CI of cardiovascular changes, i.e. worse out of two same session UDIs were used. AD=autonomic dysreflexia, AIS= American spinal injury association (ASIA) impairment scale, ANOVA=analysis of variance, BPM=beats per minute, CI=confidence interval, DBP=diastolic blood pressure, HR=heart rate, SBP=systolic blood pressure, SCI=spinal cord injury, UDI=urodynamic investigation.
